# Nematicidal effects of *Photorhabdus*- and *Xenorhabdus*-derived compounds against plant-parasitic nematodes: a systematic review and meta-analysis

**DOI:** 10.3389/fpls.2026.1891050

**Published:** 2026-07-13

**Authors:** Ignacio Vicente-Díez, Alan Zamorano, Michele Perazzolli, Carlos Castaneda-Alvarez

**Affiliations:** 1 Center Agriculture Food Environment (C3A), University of Trento, San Michele all’Adige, Italy; 2 Facultad de Ciencias Agronómicas, Universidad de Chile, Santiago, Chile

**Keywords:** bioactive compounds, biocontrol, nematode management, new biopesticides, entomopathogenic nematode symbionts

## Abstract

Plant-parasitic nematodes (PPNs) are major agricultural pests that cause substantial yield losses worldwide. However, developing sustainable alternatives to synthetic nematicides remains a critical challenge. Bacterial symbionts of entomopathogenic nematodes, particularly species of *Photorhabdus* and *Xenorhabdus*, have emerged as promising sources of bioactive compounds with nematicidal activity. Despite increasing experimental evidence, the overall effectiveness of *Photorhabdus* spp. and *Xenorhabdus* spp. and their nematicidal compounds remains to be assessed. Here, we conducted a systematic review and a meta-analysis to quantify the nematicidal effects of *Photorhabdus*- and *Xenorhabdus*-derived compounds against PPNs and characterize current experimental trends. Studies were identified from two databases (Web of Science Core Collection and Scopus) and screened according to PRISMA guidelines. A total of 27 studies were included in the qualitative analysis, and 12 of them provided appropriate data for quantitative analysis. The available literature is strongly biased toward *in vitro* assays targeting *Meloidogyne* spp., particularly *M. incognita*, and predominantly evaluates cell-free supernatants as bioactive preparations. Bacterial-derived compounds showed a positive effect on PPN suppression. The effect size was statistically significant under *in vitro* conditions, whereas *in vivo* studies showed lower effects with confidence intervals overlapping zero, indicating a non-significant trend toward nematode suppression. Our findings provide quantitative evidence that *Photorhabdus*- and *Xenorhabdus*-derived compounds have consistent nematicidal potential against PPNs. Future research should focus on expanding taxonomic coverage and improving experimental reproducibility to better assess their effectiveness under field conditions.

## 1 Introduction

Plant-parasitic nematodes (PPNs) are among the most damaging pests in agroecosystems, affecting important crops worldwide and causing global annual losses (Singh et al., 2013; Opdensteinen et al., 2024; Meel and Saharan, 2025). Symptoms of PPNs are often masked by other biotic and abiotic constraints on plant growth, and their impacts are frequently underestimated (Mandal et al., 2021), suggesting that its true economic and agronomic significance may be even greater than currently recognized. Effective PPN management requires a multifaceted strategy focused on maintaining population densities below economically damaging thresholds (Barker and Koenning, 1998; Jones et al., 2013). Common management methods include planting resistant crop varieties (Phani et al., 2024), rotating crops (Chen and Tsay, 2006; Schumacher et al., 2020; Fullana et al., 2023), incorporating soil amendments or cover crops (Oka, 2010; Zhang et al., 2025), and applying chemical or biological pesticides (Desaeger et al., 2020; Meel and Saharan, 2025).

The use of conventional nematicides has progressively declined in recent decades due to increasing regulatory restrictions driven by concerns over their environmental persistence and potential risks to human health (Desaeger et al., 2020; Burns et al., 2023). Stringent legislative revisions have reduced the use of synthetic nematicides, and only a limited number of chemical pesticides are currently authorized in the European Union, such as metam-sodium, dazomet, fosthiazate, and oxamyl, with increasing restrictions or regional revocations (Sasanelli et al., 2021). Consequently, there has been renewed interest in biological control strategies and natural products as sustainable alternatives to synthetic nematicides (Oka, 2020), with particular emphasis on agents capable of overcoming the structural defenses of PPNs (e.g., their protective multilayered cuticle, which limits the penetration and efficacy of many chemical agents during short-term exposure) and maintaining activity in the soil environment.

Among biological pesticides against PPNs, products derived from *Photorhabdus* spp. and *Xenorhabdus* spp. have received increasing attention due to their ability to produce a wide range of compounds with nematicidal properties (Bode, 2009; Hazir and Bode, 2024). *Photorhabdus* spp. and *Xenorhabdus* spp. are entomopathogenic symbionts of nematodes belonging to the genera *Heterorhabditis* and *Steinernema*, respectively (Shi and Bode, 2018). Several studies have reported nematicidal activity of *Photorhabdus* spp. and *Xenorhabdus* spp. against a variety of PPN species under laboratory, greenhouse, and field conditions (Sayedain et al., 2019; Abebew et al., 2022). However, the effects of entomopathogenic symbionts vary across studies, reflecting differences in bacterial strains, metabolite composition, environmental conditions, and soil properties (Kusakabe et al., 2022; Krithika et al., 2024; Deeikshana et al., 2025). This variability makes it difficult to directly compare results and assess the consistency of the observed effects across experimental systems.

Despite this growing evidence, the overall effectiveness of entomopathogenic symbionts and their derived compounds lacks a quantitative basis. A meta-analysis is therefore needed to integrate available data, quantify effect sizes, and identify sources of variability among studies on entomopathogenic symbionts against PPNs. Here, we present a systematic review and meta-analysis of the nematicidal activity of *Photorhabdus*- and *Xenorhabdus*-derived compounds against PPNs. By compiling data from independent studies conducted under diverse experimental conditions, we aimed to (i) characterize current research trends and experimental approaches, (ii) quantify the overall magnitude of the nematicidal effects through meta-analysis; (iii) summarize current knowledge on the compounds produced by these bacteria; and (iv) highlight possible future research directions on *Photorhabdus* spp. and *Xenorhabdus* spp. to fill key gaps that limit their application under field conditions.

## 2 Materials and methods

### 2.1 Literature search and data collection

The selection of published studies followed two systematic review frameworks: the research question was formulated using the PICO framework (Miller and Forrest, 2001), and the study selection process followed the PRISMA 2020 guidelines (Page et al., 2021) (**Supplementary Figure 1**). The literature search was conducted in September 2025 and updated in December 2025. Records were identified through the Web of Science Core Collection (WoS) and SCOPUS databases. Additional records were identified through ResearchGate. Studies were searched in these three databases using the following keywords and Boolean operators: (*Photorhabdus* OR *Xenorhabdus*) AND (extract OR filtrate OR broth OR culture filtrate OR culture supernatant OR supernatant* OR cell-free OR fermented medium OR metabolite* OR secondary metabolite* OR toxin* OR compound* OR natural product* OR bioactive compound* OR volatile organic compound*) AND (nematod* OR plant-parasitic nematode* OR *Meloidogyne* OR *Heterodera* OR *Globodera* OR *Pratylenchus* OR *Xiphinema* OR *Radopholus* OR *Ditylenchus* OR *Tylenchulus*) AND (nematicidal OR mortality OR suppression OR infectivity OR reproduction OR egg hatching OR nematode mortality* OR J2). In this search string, ‘J2’ refers to second-stage juveniles, a standard term in plant nematology commonly used for the infective juvenile stage evaluated in nematicidal bioassays.

The literature search covered the entire period available in each database up to the date of the final query. Only peer-reviewed original research articles published in English were considered. Records retrieved from different databases were combined, and duplicate entries were removed. Bibliographic information, including authors, title, year of publication, journal, and DOI, was extracted for all unique records. A total of 283 records were screened based on title and abstract, and 22 review papers and 233 studies that did not target PPNs were excluded from further analyses. The remaining 27 papers were assessed for full-text eligibility, and all were retained for qualitative analysis (**Table 1**). Among them, 12 studies provided sufficient quantitative data for inclusion in the meta-analysis, for a total of 566 effect sizes, such as comparisons of different treatments, concentrations, or time points (**Supplementary Figure 1**). Basic study-quality information, including the presence of replication, negative or positive controls, and reported measures of variability, was recorded during data extraction and considered when assessing the reliability and interpretability of the quantitative synthesis. Quantitative data were extracted directly from the main text or tables following approaches used in previous meta-analyses (Gurevitch et al., 2018; Karimi et al., 2020; Serrão et al., 2024; Hendra et al., 2026).

**Table 1:** Overview of studies included in the systematic review evaluating the effects of bacterial symbionts against plant-parasitic nematodes.

System	Nematode target	References
*In vitro* (nematode mortality)	*Meloidogyne* spp.	Samaliev et al., 2000; **Fallon et al., 2004**; **El-Deen et al., 2014**; **Sayedain et al., 2019**; **Abebew et al., 2022**; Kusakabe et al., 2022; Li et al., 2023; Li et al., 2024; Arumugam et al., 2024; Deeikshana et al., 2024; **Deeikshana et al., 2025**; **Gondal et al., 2024**; González-Trujillo et al., 2024; Kim et al., 2024; **Krithika et al., 2024**; Larkin et al., 2025
	Others	Kusakabe et al., 2022; **Kim et al., 2024**; Larkin et al., 2025
*In vivo* (nematode reproduction)	*Meloidogyne* spp.	Hu et al., 1999; Fallon et al., 2004; Zakaria et al., 2013; **Kepenekci et al., 2016**; Kepenekci et al., 2018; Orozco et al., 2016; Bi et al., 2018; **Caccia et al., 2018**; **El-Deen et al., 2019**; Kusakabe et al., 2022; Li et al., 2023; González-Trujillo et al., 2024; Kim et al., 2024; Deeikshana et al., 2025
	Others	Hu et al., 1999; **Caccia et al., 2018**

Studies are categorized based on experimental approach, including *in vitro* assays assessing nematode mortality and *in vivo* assays evaluating nematode reproduction. The 12 studies providing sufficient quantitative data for inclusion in the meta-analysis are highlighted in bold.

Data were categorized at two complementary levels: experimental system and response variable (**Table 2**). Regarding the experimental system, *in vitro* assays were defined as experiments conducted under laboratory culture conditions, in the absence of a host plant, in which nematodes or eggs were directly exposed to bacterial compounds. The response variables recorded for *in vitro* assays included egg hatching, defined as the emergence of juveniles from eggs; J2 mortality, defined as the mortality of second-stage juveniles; and nematode population, defined as the number of nematodes recovered from the experimental system. *In vivo* assays were defined as experiments conducted with plants under greenhouse, growth chamber, or field conditions, in which treatment effects were evaluated within a plant–nematode system. The response variables recorded for *in vivo* assays included gall severity, defined as the intensity of root galling; nematode population, defined as the number of nematodes recovered from soil, roots, or plant tissues; nematode reproduction, defined as direct measures of nematode multiplication; and reproduction factor, defined as a relative measure of population increase, usually expressed as the ratio between final and initial nematode population.

**Table 2:** Summary of publications considered in the meta-analysis. .

Reference	*In vitro*			*In vivo*			
	Egg hatching	Mortality J2	Nematode population	Gall severity	Nematode population	Nem. Rep.	Nem. Rep. factor
Aatif et al., 2021				*		*	
**Abebew et al., 2022**		*					
**Arumugam et al., 2024**	*	*					
**Caccia et al., 2018**				*		*	*
**Deeikshana et al., 2025**		*					
**El-Deen et al., 2014**		*					
**El-Deen et al., 2019**				*	*	*	
**Fallon et al., 2004**	*				*	*	
**Gondal et al., 2024**	*						
González-Trujillo et al., 2024		#		#		#	
**Kepenekci et al., 2016**						*	
Kepenekci et al., 2018				*			
**Kim et al., 2024**		#			*	*	
**Krithika et al., 2024**		*					
Larkin et al., 2025		*					
Samaliev et al., 2000			*				
**Sayedain et al., 2019**	*	*					
Tülek et al., 2018							
Bi et al., 2018							
Deeikshana et al., 2024							
Hu et al., 1999							
Kusakabe et al., 2022							
Kusakabe et al., 2023							
Li et al., 2023							
Li et al., 2024							
Orozco et al., 2016							
Zakaria et al., 2013							

Asterisks indicate assessed parameters with data suitable for quantitative meta-analysis. Hashtags indicate assessed parameters that were not suitable for meta-analysis because the data were transformed or because the corresponding control values were not reported. The 27 publications included in the systematic review are listed, and the 12 studies providing quantitative data for meta-analysis are highlighted in bold.

For the quantitative meta-analysis, we focused on the response variables most consistently reported across studies, primarily J2 mortality under *in vitro* assays and nematode reproduction under *in vivo* assays. When numerical values were reported only in graphical form and raw data were unavailable, they were extracted using WebPlotDigitizer (https://automeris.io/wpd/) as reported by Serrão et al. (2024). Extracted variables included sample size, mean mortality, reproduction values, and measures of variability (standard deviation, standard error, or confidence intervals). In studies assessing multiple concentrations or exposure times, the highest tested concentration and the final assessment time point were selected to ensure maximum efficacy for each experiment. This approach was adopted to standardize effect estimation across heterogeneous experimental designs, although it may bias effect sizes toward maximum responses. This limitation was considered in the interpretation of the results. Taxonomic nomenclature of bacterial symbionts was recorded as originally reported in each study.

### 2.2 Effect size calculation and statistical analysis

Effect sizes were calculated as the log response ratio (LRR) defined as:


$$\text{LRR}=\ln\left(\frac{\text{Vt}}{\text{Vc}}\right)$$

where *V_t_* is the mean response of the treatment group (exposure to *Photorhabdus*- or *Xenorhabdus*-derived products) and *V_c_* is the mean response of the corresponding control group (Serrão et al., 2024). For mortality data (*in vitro* assays), positive LRR values indicate increased nematode mortality relative to controls. For reproduction data (*in vivo* assays), effect sizes were sign-transformed (i.e., multiplied by −1), and positive values indicate reduced nematode reproduction relative to controls.

When necessary, standard deviations (SD) were reconstructed from reported standard errors (SE) as:


SD=SE·√n

Whenever possible, n corresponded to the number of independent experimental replicates. Sampling variances for LRR values were calculated using the delta-method approximation for response-ratio data (Hedges et al., 1999; Viechtbauer, 2010).

Because several publications reported multiple treatment–control comparisons, the extracted effect sizes were not all treated as statistically independent observations in the primary meta-analysis. For the main quantitative synthesis, a conservative study-level approach was adopted. Separate datasets were prepared for *in vitro* mortality and *in vivo* reproduction assays. Within each publication and analytical domain, the highest tested concentration and the longest exposure time were selected. When multiple eligible comparisons remained within the same publication, for example, because different bacterial species, strains, or treatment arms were tested under the same conditions, these comparisons were averaged to obtain a single study-level effect size. This approach was used to avoid giving disproportionate weight to publications reporting many treatment arms.

Separate random-effects meta-analyses were conducted for *in vitro* and *in vivo* datasets using the restricted maximum likelihood (REML) estimator according to (Viechtbauer, 2010). Between-study heterogeneity was quantified using the I^2^ statistic and τ^2^ estimates derived from the random-effects models according to Higgins et al. (2003). The I^2^ statistic represents the proportion of total observed variability in effect sizes that is attributable to true between-study heterogeneity rather than sampling error, whereas τ^2^ estimates the absolute between-study variance in the underlying true effects. Higher values of I^2^ and τ^2^ therefore indicate greater inconsistency among studies and suggest that effect sizes differ beyond what would be expected from random sampling variation alone. Effect sizes were weighted by the inverse of their sampling variance, giving greater weight to more precise estimates as reported by Borenstein et al. (2021).

To evaluate the potential influence of effect-size dependency and to explore sources of heterogeneity, exploratory multilevel meta-analytic models were fitted using the full set of eligible treatment–control comparisons. In these models, study identity was included as a random factor to account for the non-independence of multiple effect sizes extracted from the same publication. This approach is recommended when several dependent effect sizes are derived from the same study (Van den Noortgate et al., 2013; Assink and Wibbelink, 2016). Exploratory moderator analyses were conducted whenever sufficient data were available. The moderators evaluated included bacterial genus, bacterial species, preparation type, target nematode identity, target nematode group, feeding habit, and exposure duration. These analyses were considered exploratory because of the limited number of independent studies contributing to each analytical domain.

Sensitivity analyses were performed to assess the influence of individual studies and of studies with unusually large LRR values associated with near-zero responses in the control group. These included leave-one-out analyses, influence diagnostics, and additional models excluding selected influential studies. These sensitivity analyses were used to evaluate the robustness of the pooled estimates but did not replace the primary analysis, including all eligible studies.

Effect sizes were visualized using combined plots representing the *in vitro* (nematode mortality assessed under laboratory culture conditions), *in vivo* (nematode reproduction assessed on plants under greenhouse or field conditions), and global meta-analytic model (both *in vitro* and *in vivo* datasets). Potential small-study effects were evaluated through visual inspection of funnel plots and Egger’s regression test (Egger et al., 1997), although these analyses were interpreted cautiously due to the small number of independent studies. All statistical analyses were performed in *R version 4.3.2*.

## 3 Results

### 3.1 General summary on nematicidal effects of *Photorhabdus* spp. and *Xenorhabdus* spp.

The studies included in the systematic review (27 publications) exhibited considerable variation in experimental systems (*in vitro* or *in vivo*), target nematodes, and response variables (e.g., egg hatching, gall severity, J2 mortality, nematode population, and nematode reproduction) assessed (**Tables 1**, **2**). Experimental systems were primarily *in vitro* assays, which accounted for the largest proportion of studies (> 60%) and predominantly evaluated early life stages, particularly nematode J2 mortality and egg hatching. In contrast, *in vivo* experiments were less frequent and focused mainly on plant-level outcomes, including gall severity, nematode population density, and nematode reproduction. The temporal distribution of publications showed a gradual increase in research activity over time, indicating growing interest in the nematicidal potential of these bacterial symbionts (**Supplementary Figure 2**).

**Figure 1:**
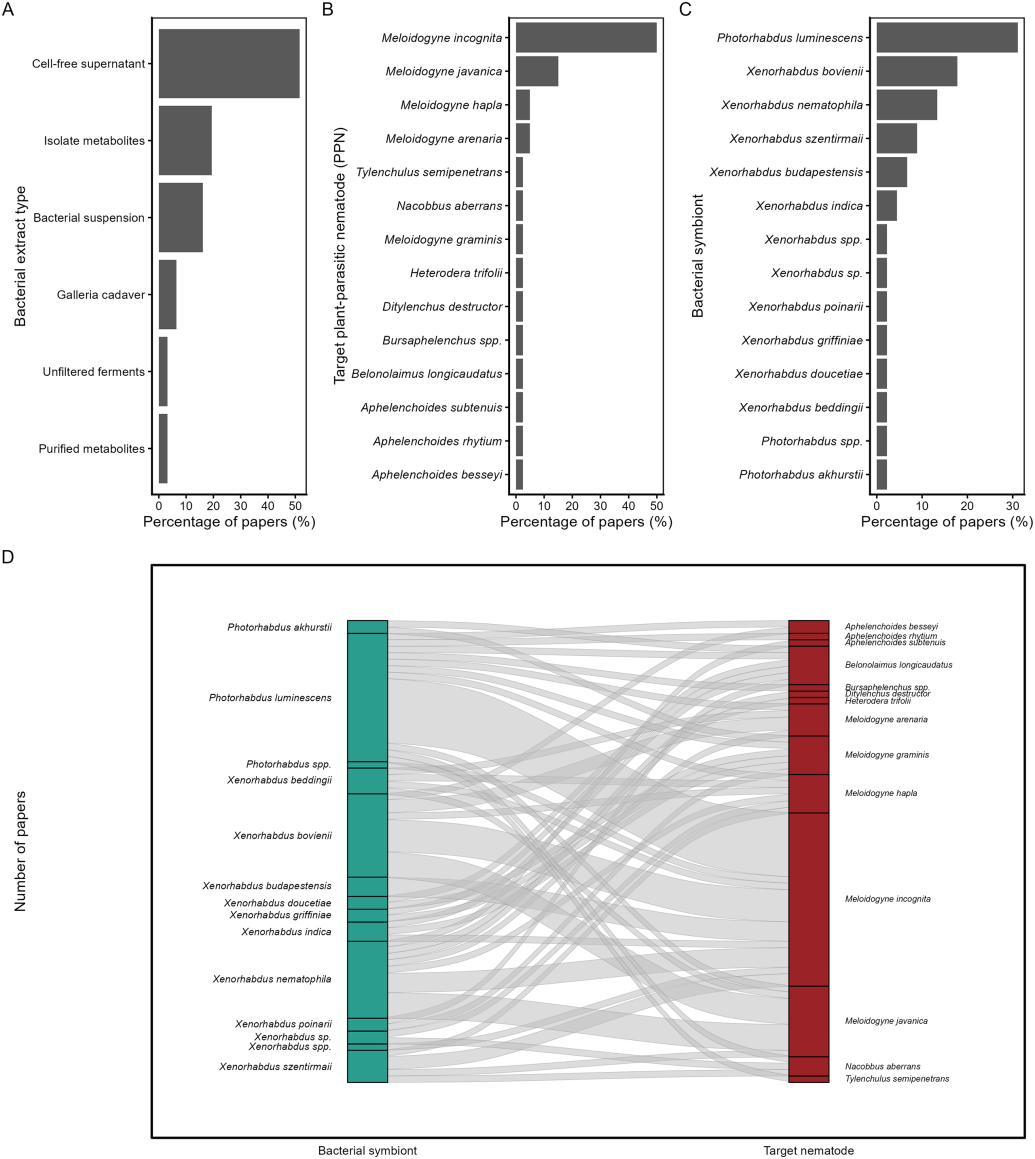
Summary of experimental systems, bacterial symbionts, and plant-parasitic nematode targets reported in the studies included in the systematic review. Frequency of bacterial extract types used across studies **(A)**. Frequency of target plant-parasitic nematode taxa **(B)**. Frequency of bacterial symbiont species investigated **(C)**. Alluvial diagram showing the relationships between bacterial symbionts and target nematodes across the studies **(D)**.

Regarding the type of bacterial derived bioproducts evaluated, cell-free supernatants were the most commonly tested preparations, representing approximately half of the studies included in the dataset (**Figure 1A**). Other preparation types—such as crude metabolites, bacterial suspensions, and cadaver-derived materials—were less frequently assessed. Studies targeting *Meloidogyne* spp. represented the majority of PPNs across both *in vitro* and *in vivo* experiments (**Figure 1B**). Among the target nematodes, *Meloidogyne incognita* was the most frequently studied species, whereas other nematode taxa, including the genera *Heterodera*, *Ditylenchus*, and *Aphelenchoides*, were rarely investigated. Moreover, the taxonomic distribution of bacterial symbionts was uneven, with *Photorhabdus luminescens* accounting for the largest proportion of study records (31.1%), followed by *Xenorhabdus bovienii* (17.8%) and *X. nematophila* (13.3%), while other species were investigated only occasionally (**Figures 1C, D**).

### 3.2 *Photorhabdus*- and *Xenorhabdus*- derived compounds with nematicidal effects

Compounds derived from *Photorhabdus* spp. and *Xenorhabdus* spp. reported in the publications included in the systematic review comprise a chemically diverse set of metabolites; a total of 12 nematicidal compounds were identified across the dataset (**Table 3**). A substantial proportion of the reported compounds are associated with *Xenorhabdus* spp. (58%), including several secondary metabolites synthesized through non-ribosomal peptide synthase (NRPS), polyketide synthase (PKS), or hybrid NRPS–PKS pathways, such as fabclavines, rhabdopeptides, and xenocoumacins. In contrast, compounds linked to *Photorhabdus* spp. (33%) are more frequently derived from shikimate-related pathways, including trans-cinnamic acid, phenylpropionic acid, volatile organic compounds (e.g., dimethyl disulfide), and polyphenolic compounds (e.g., stilbenes). A small subset of compounds (8%) was reported to be associated with both genera, such as indole. Several compounds were also linked to primary metabolic pathways, including malonate, thymine, sedoheptulose-7-phosphate, and prenylated purines. The level of chemical characterization varied among studies, ranging from the identification of specific biosynthetic gene clusters to broader associations with metabolic pathways.

**Table 3:** Main nematicidal compounds produced by *Xenorhabdus* and *Photorhabdus* spp., including their chemical classes, biosynthetic origin, and associated gene clusters.

Compound	Chemical class	Bacterial genus	Biosynthetic pathway	Biosynthetic genes	References
Fabclavines	Hybrid NRPS–PKS peptides	*Xenorhabdus*	Hybrid NRPS–PKS pathway	*fclA–fclP* (fabclavin BGC)	Bi et al., 2018; Abebew et al., 2022; Deeikshana et al., 2024; Krithika et al., 2024
Rhabdopeptides	Nonribosomal peptides	*Xenorhabdus*	NRPS pathway	*rhpA–rhpE*	Bi et al., 2018; Abebew et al., 2022; Deeikshana et al., 2024
Xenocoumacins	Polyketide–peptide hybrids	*Xenorhabdus*	Hybrid PKS–NRPS pathway	*xcnA–xcnN*	Abebew et al., 2022; Deeikshana et al., 2024; Krithika et al., 2024
trans-Cinnamic acid (t-CA)	Phenylpropanoid	*Photorhabdus*	Shikimate-derived metabolism	*aroA–aroE* and downstream decarboxylases	Kusakabe et al., 2022, 2023
Phenylpropionic acid (PPA)	Aromatic acid	*Photorhabdus*	Shikimate / phenylalanine-derived metabolism	*aro* genes and tailoring enzymes	Kusakabe et al., 2022, 2023
Malonate	Dicarboxylic acid	*Xenorhabdus*	Central metabolism (PKS precursor)	*matA–matC*, acetyl-CoA carboxylase	Deeikshana et al., 2024
Indole	Alkaloid	*Photorhabdus*/ *Xenorhabdus*	Tryptophan catabolism	*tnaA* (tryptophanase)	Hu et al., 1999; Orozco et al., 2016; Kusakabe et al., 2022
Thymine	Pyrimidine nucleobase	*Xenorhabdus*	Nucleotide metabolism	*thyA*, *thyX*	Kim et al., 2024
6-(γ,γ-Dimethylallylamino)-purine	Prenylated purine	*Xenorhabdus*	Prenyltransferase-mediated pathway	*ipt*, DMATS-like genes	Deeikshana et al., 2025
Sedoheptulose-7-phosphate	Sugar phosphate	*Xenorhabdus*	Pentose phosphate pathway	*tktA*, *talB*	Deeikshana et al., 2025
Dimethyl disulfide (DMDS)	Volatile sulfur compound	*Photorhabdus*	Sulfur amino acid catabolism	*metC*, *metE*, *mgl*	Li et al., 2024
Stilbenes (ST)	Polyphenolic stilbenes	*Photorhabdus*	Type III polyketide synthase pathway	*stlA*, *stlB*	Hu et al., 1999; Orozco et al., 2016

BGC, biosynthetic gene cluster; DMATS, dimethylallyltryptophan synthase; DMDS, dimethyl disulfide; NRPS, nonribosomal peptide synthase; PKS, polyketide synthase; PPA, phenylpropionic acid; ST, stilbenes; t-CA, trans-cinnamic acid); and genes involved in central metabolic pathways (e.g., *aro, tnaA, thyA/thyX, ipt, tktA, talB, matA–matC, metC, metE*, and *mgl*).

### 3.3 Meta-analysis on nematicidal effects of *Photorhabdus* spp. and *Xenorhabdus* spp.

**Figure 2:**
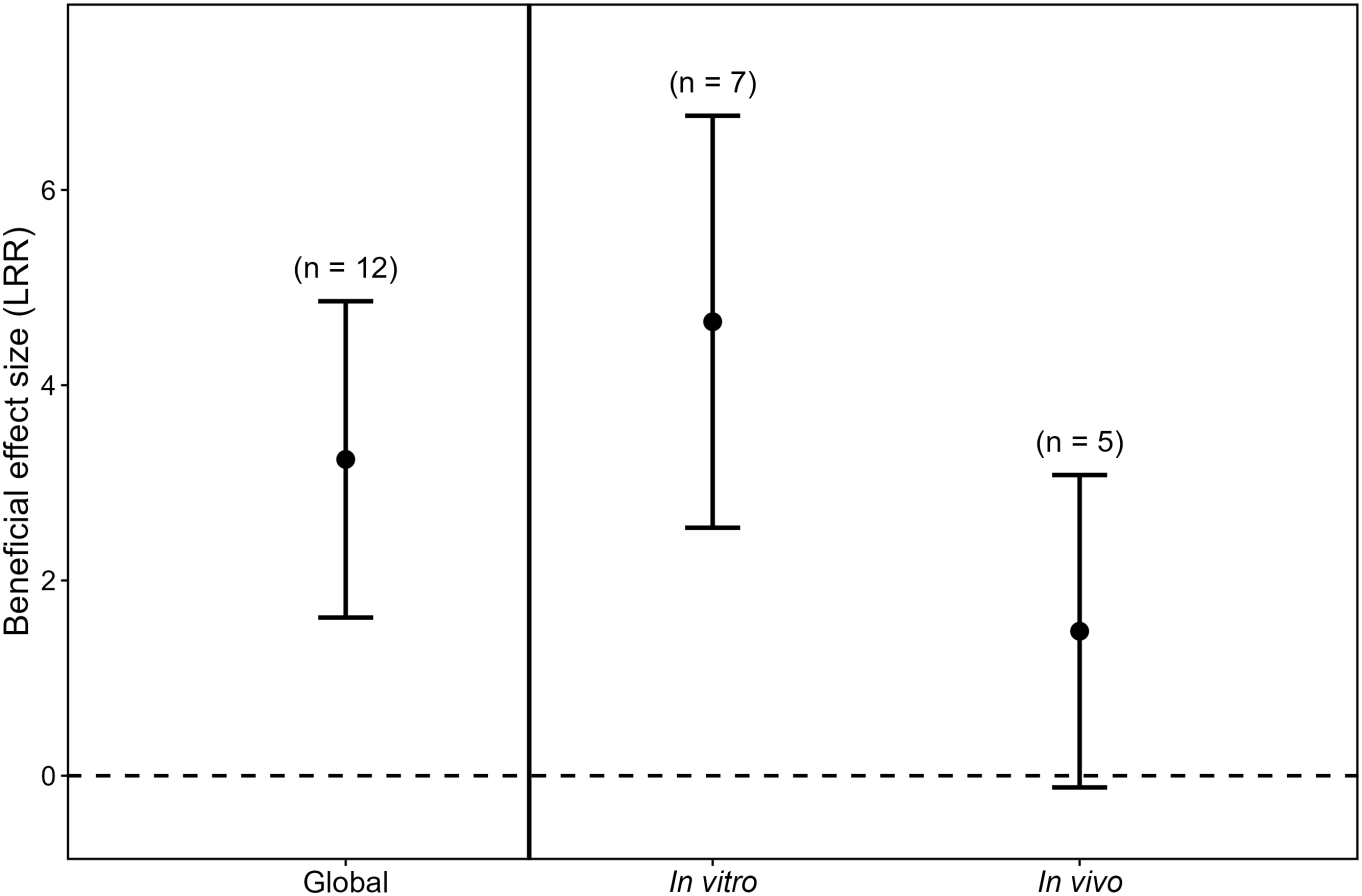
Effect sizes for the suppression of plant-parasitic nematodes by *Photorhabdus*- and *Xenorhabdus*-derived compounds across experimental conditions. Points represent mean log response ratios (LRR) and error bars indicate 95% confidence intervals for the global dataset (12 publications), *in vitro* assays on nematode mortality (7 publications), and *in vivo* assays on nematode reproduction (5 publications). The dashed horizontal line indicates no effect (LRR = 0).

The quantitative analysis revealed a beneficial effect of *Photorhabdus* and *Xenorhabdus*-derived compounds on the suppression of PPNs (**Figure 2**). The global meta-analysis, including 12 publications (7 *in vitro* and 5 *in vivo*), showed a positive effect size (LRR = 3.24, 95% confidence interval: 1.6 to 4.9, p< 0.001), indicating a beneficial effect on nematode suppression across studies. When analyzed separately, *in vitro* assays (7 publications) exhibited a high positive effect size (LRR = 4.6, 95% confidence interval: 2.5 to 6.7, p< 0.001) and confidence intervals that remained above zero, supporting the nematicidal effects of *Photorhabdus*- and *Xenorhabdus*-derived compounds. Moreover, *in vivo* experiments (5 publications) showed a positive sign-transformed effect size (LRR = 1.48, 95% confidence interval: -0.1 to 3.1) and wide confidence intervals overlapping zero, indicating a non-significant trend toward nematode suppression (p = 0.07).

Substantial heterogeneity was detected in both *in vitro* assays (I^2^ = 99.1%, τ^2^ = 7.10) and *in vivo* assays (I^2^ = 97.6%, τ^2^ = 3.15), indicating marked variability in effect sizes among studies. Therefore, the pooled estimates should be interpreted cautiously as evidence of overall trends rather than as precise estimates of homogeneous treatment effects.

To evaluate the potential influence of effect-size dependency, exploratory multilevel meta-analytic models were fitted using the full set of eligible treatment–control comparisons and including study identity as a random factor. The *in vitro* multilevel model included 349 effect sizes from 7 studies and showed a significant positive effect (LRR = 4.16, 95% CI: 2.65 to 5.66, p< 0.001), consistent with the conservative study-level model. The *in vivo* multilevel model included 30 effect sizes from 5 studies and also showed a positive effect (LRR = 0.93, 95% CI: 0.30 to 1.55, p< 0.05).

Exploratory moderator analyses were conducted to investigate potential sources of heterogeneity in the *in vitro* dataset. Exposure duration significantly explained part of the observed heterogeneity (QM = 67.1, p< 0.001). In contrast, bacterial genus (p = 0.502), bacterial species (p = 0.277), target nematode identity (p = 0.781), and target nematode group (p = 0.834) were not significant moderators. These results suggest that exposure duration contributed to the variability among *in vitro* effect sizes, although substantial unexplained heterogeneity remained.

Sensitivity analyses showed that the largest *in vitro* effect sizes were associated with studies reporting near-zero mortality in the negative control. Excluding Sayedain et al. (2019) reduced the pooled *in vitro* estimate from LRR = 4.65 to LRR = 4.07, while excluding both Sayedain et al. (2019) and El-Deen et al. (2014) reduced the estimate to LRR = 3.08 and decreased heterogeneity from I^2^ = 99.1% to I^2^ = 85.8%. Importantly, the effect remained positive and statistically significant in all sensitivity analyses. These results indicate that influential studies contributed to the magnitude of the pooled effect and to heterogeneity, but did not fully account for the overall positive direction of the *in vitro* response.

Potential small-study effects were assessed by visual inspection of funnel plots and Egger’s regression test. Funnel plot inspection did not indicate a clear asymmetrical pattern, although this assessment should be interpreted cautiously because of the limited number of studies included in the global model. Consistently, Egger’s regression test did not detect significant funnel plot asymmetry in the global dataset (z = −0.4567, p = 0.6479), suggesting no statistical evidence of small-study effects.

## 4 Discussion

Our systematic review revealed a clear imbalance in the evidence on nematicidal capacity of compounds produced by entomopathogenic bacteria against PPNs, characterized by a limited number of bacterial species (mainly *Photorhabdus* spp. and *Xenorhabdus* spp.), a strong emphasis on cell-free supernatants, and a narrow taxonomic range of target PPNs (mainly *Meloidogyne* spp.), as previously reported (Sayedain et al., 2019; González-Trujillo et al., 2024). This taxonomic bias reflects the global economic importance of *Meloidogyne* spp (Abebew et al., 2022; Arumugam et al., 2024) and the widespread availability of established laboratory bioassays for this genus, limiting the generalizability of current findings to other PPN taxa (Kim et al., 2024; Larkin et al., 2025). This is particularly relevant for economically important groups such as cyst nematodes and lesion nematodes, which remain comparatively underrepresented in the current literature. Moreover, a clear imbalance was observed in the type of experimental systems used, with most studies conducted under *in vitro* conditions, whereas *in vivo* and field-based experiments remain comparatively scarce, potentially limiting the extrapolation of these findings to real agricultural settings.

Beyond taxonomic bias, a clear methodological pattern also emerged. Cell-free supernatants were mainly used to assess the nematicidal activity of *Photorhabdus* spp. and *Xenorhabdus* spp., corroborating that most studies have evaluated secreted metabolites rather than living bacterial cells (Abebew et al., 2022; Arumugam et al., 2024; Kim et al., 2024). These cell-free preparations have demonstrated strong suppressive effects on nematode reproduction and infection (Kepenekci et al., 2018), in agreement with the extensive secondary metabolite repertoire of *Xenorhabdus* spp. and *Photorhabdus* spp (Abebew et al., 2022; Krithika et al., 2024). However, the variability in effect sizes across studies indicated that treatment efficacy is possibly influenced by the bacterial strain, metabolite composition, and experimental conditions (Arumugam et al., 2024; González-Trujillo et al., 2024).

Although a wide range of bioactive compounds has been reported, including fabclavines, rhabdopeptides, xenocoumacins, trans-cinnamic acid, phenylpropionic acid, indole, and stilbenes (El-Deen et al., 2014, 2019; Abebew et al., 2022; Gondal et al., 2024; Deeikshana et al., 2025), their relative contribution to nematicidal activity cannot be quantitatively compared due to methodological differences. Consequently, the analysis of compounds should be interpreted as a general overview rather than a quantitative assessment. From an applied perspective, these bacterial compounds show potential uses in integrated pest management strategies, in combination with organic amendments or reduced doses of conventional nematicides (Fallon et al., 2004; Kepenekci et al., 2016; Bi et al., 2018a; Kusakabe et al., 2023; Krithika et al., 2024). However, the efficacy of *Photorhabdus*- and *Xenorhabdus*-derived compounds under field conditions remains insufficiently characterized, and factors affecting metabolite stability and persistence should be further investigated, such as soil physicochemical properties, microbial interactions, and compound degradation (Zakaria et al., 2013; Kepenekci et al., 2016).

Our meta-analysis indicated an overall positive effect of *Photorhabdus*- and *Xenorhabdus*-derived compounds on PPNs suppression. Effect sizes were numerically higher *in vitro* (LRR = 4.6) than *in vivo* (LRR = 1.48). These differences should be interpreted cautiously because *in vitro* mortality and *in vivo* reproduction represent biologically and experimentally distinct endpoints, and because both datasets showed very high heterogeneity. Therefore, the pooled estimates should be understood as evidence of an overall beneficial trend rather than as precise estimates of a uniform treatment response. The non-significant effect *in vivo* suggests a strong influence of environmental and biological factors, which may reduce the consistency of nematicidal effects under more realistic conditions. In addition, some limitations of the present study should be considered. First, the meta-analysis was based on a limited number of studies and heterogeneous experimental designs. Second, selecting the highest concentration and longest exposure time may have biased effect size estimates toward maximum responses. Third, taxonomic inconsistencies in bacterial identification may also contribute to variability among studies. Future research should therefore address these limitations by expanding taxonomic coverage of both bacterial symbionts and target nematode species, increasing *in vivo* and field-based studies with standardized designs, linking metabolite profiles to nematicidal outcomes, and investigating the stability of bioactive compounds under realistic soil conditions. Particular attention should be given to formulation technologies that improve the delivery, protection, and persistence of bacterial-derived compounds in soil. It will also be important to determine how soil physicochemical properties, native microbial communities, microbial competition, and environmental degradation affect metabolite stability, bioavailability, and nematicidal activity under agronomically realistic conditions.

## 5 Conclusions

This systematic review and meta-analysis suggest that bacterial compounds derived from *Photorhabdus* and *Xenorhabdus* exert consistent nematicidal effects against PPNs. Despite the diversity of experimental systems and metabolites reported across studies, the overall effect was consistently positive. The results also reveal important limitations in the current evidence, including a taxonomic bias toward *Meloidogyne* spp., a predominance of experimental assays under controlled conditions, and a focus on a limited number of bacterial symbionts and preparation types. These constraints limit the generalizability of the available data and hinder the extrapolation of results to field conditions. While a wide range of bioactive compounds has been reported, their specific contributions to the observed effects remain difficult to disentangle across studies. This limitation highlights the need for more integrative approaches that link metabolite profiles with nematicidal outcomes and underlying mechanisms. Future research should therefore prioritize improving the ecological relevance, taxonomic coverage of entomopathogenic bacteria, and methodological consistency of experimental approaches to better assess the effectiveness of these bacterial compounds under field conditions. Addressing these limitations will be essential to provide a robust quantitative basis for advancing the development and application of *Photorhabdus*- and *Xenorhabdus*-derived compounds as sustainable tools for PPN management.

## Data Availability

The raw data supporting the conclusions of this article will be made available by the authors, without undue reservation.
