# Supplementary material for: Nematicidal effects of Photorhabdus- and Xenorhabdus-derived compounds against plant-parasitic nematodes: a systematic review and meta-analysis
Source: Front Plant Sci. 2026 Jul 13;17:1891050. doi: 10.3389/fpls.2026.1891050 (PMC13402425; doi:10.3389/fpls.2026.1891050)

Supplementary material

Nematicidal effects of *Photorhabdus*- and *Xenorhabdus*-derived compounds against plant-parasitic nematodes: a systematic review and meta-analysis

I. Vicente-Díez, A. Zamorano, M. Perazzolli, C. Castaneda-Alvarez

Supplementary Figure S1. PRISMA flow diagram illustrating the identification, screening, eligibility, and inclusion of studies in the systematic review.

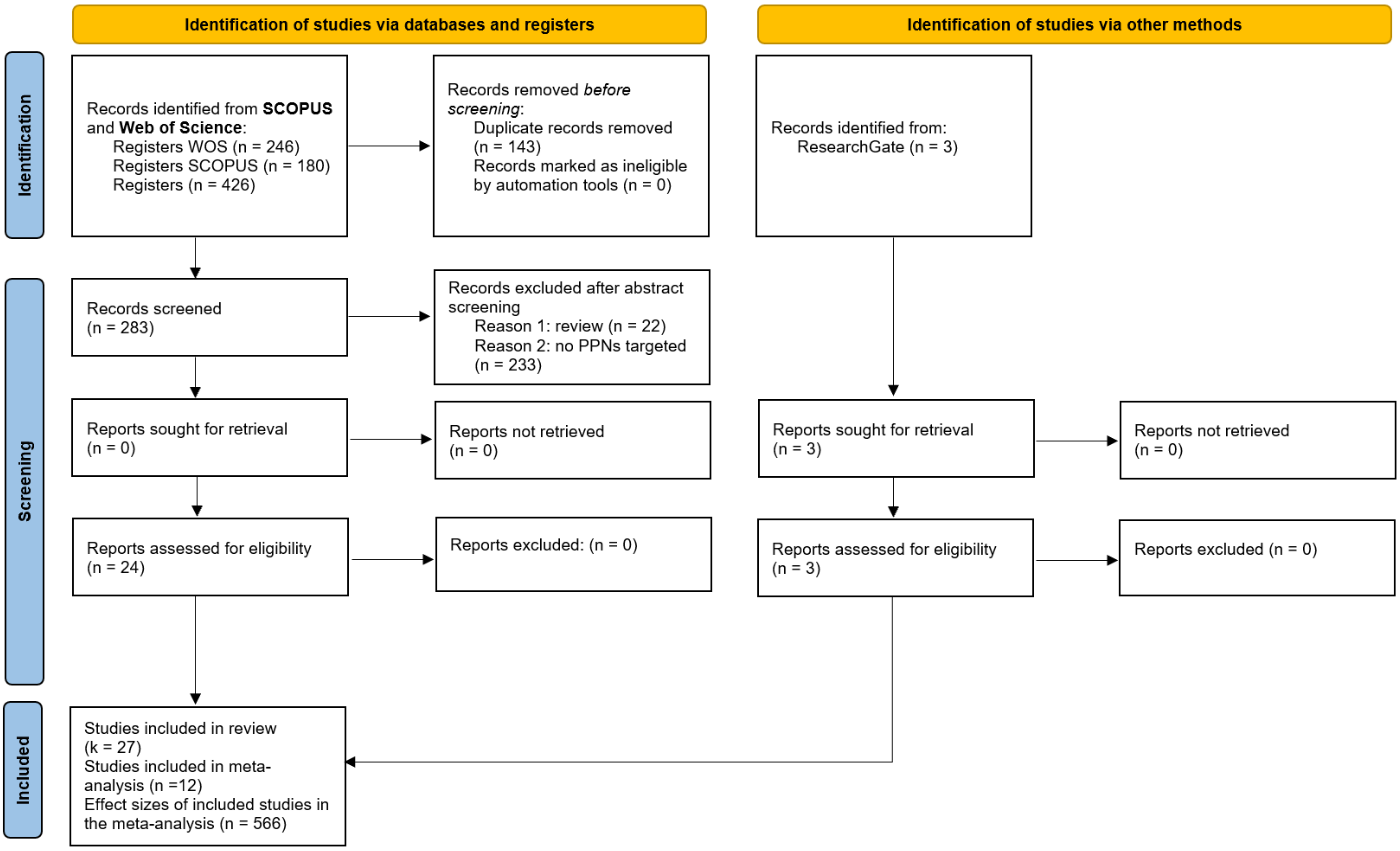

Supplementary Figure S2. Temporal distribution of the 27 publications included in the systematic review.

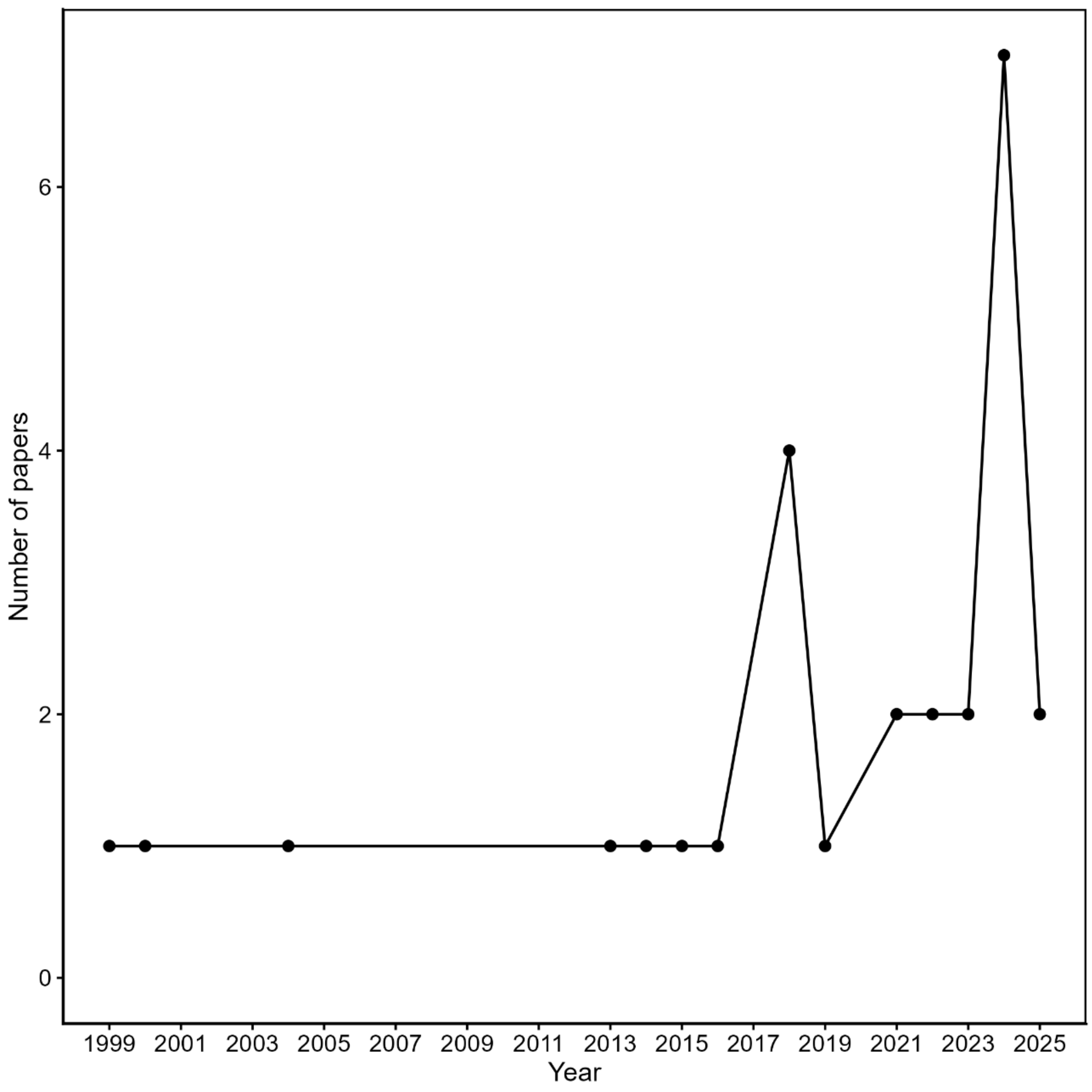

Supplement: Supplementary file 1 [file DataSheet1.pdf]
